# The effect of lactose and a prototype *Lactobacillus acidophilus* fermentation product on digestibility, nitrogen balance, and intestinal function of weaned pigs^1^

**DOI:** 10.1093/tas/txaa045

**Published:** 2020-04-17

**Authors:** Jesus A Acosta, Nicholas K Gabler, John F Patience

**Affiliations:** Department of Animal Science, Iowa State University, Ames, IA

**Keywords:** intestinal enzymes, intestinal morphology, intestinal permeability, nursery pigs, weaning stress

## Abstract

The objective of this study was to determine the effects of lactose (LA) and a prototype *Lactobacillus acidophilus* fermentation product (FP) on growth performance, diet digestibility, nitrogen (N) balance, and intestinal function of weaned pigs. Twenty-eight newly weaned pigs [approximately 21 d of age; initial body weight (BW) = 5.20 ± 0.15 kg] were housed in metabolism crates and assigned to one of four treatments (*n* = seven pigs per treatment) corresponding to a 2 × 2 factorial design: with (LA**+**; 15% inclusion) or without (LA−) LA and with (FP+) or without (FP−) the prototype FP (1 g of FP per kilogram of diet; Diamond V, Cedar Rapids, IA). Feed and water were provided ad libitum. At day 5, pigs were orally given lactulose and mannitol to assess small intestinal permeability. Fecal samples were collected on days 5–9 to determine the apparent total tract digestibility (ATTD) of dry matter (DM), gross energy (GE), and N. Total urine output and fecal samples were collected on days 10–13 to determine N retention. On day 15, all pigs were euthanized to collect intestinal lumen and tissue samples. Data were analyzed for the main effects of LA and FP and their interaction using the MIXED procedure of SAS. Lactose improved average daily feed intake (ADFI; *P* = 0.017), the ATTD of DM (*P* = 0.014), the ATTD of GE (*P* = 0.028), and N retention (*P* = 0.043) and tended to increase the butyric acid concentration in the colon (*P* = 0.062). The FP tended to increase the digestibility of N (*P* = 0.090). Neither LA nor the FP affected intestinal barrier function or inflammation markers. The interaction between LA and FP affected intestinal morphology: in the jejunum, pigs fed LA+FP− had increased villus height compared with those fed LA+FP+ and LA−FP−, whereas LA+FP+ was intermediate (interaction *P* = 0.034). At the terminal ileum, pigs fed LA−FP+ and LA+FP− had increased villus height and villus: crypt compared with those fed LA−FP−, whereas LA+FP+ was intermediate (interaction *P* = 0.007 and *P* = 0.007, respectively). In conclusion, the addition of LA brings important nutritional attributes to nursery diets by improving feed intake, digestibility of DM and GE, and the N retention of weaned pigs; however, the functional capacity of LA to improve markers of intestinal function is limited. On the other hand, the FP showed only a mild increase in the digestibility of N but a limited capacity to improve markers of intestinal function.

## INTRODUCTION

Weaning is one of the most difficult transitions in the life of a pig and greatly impacts the productivity of swine operations. Exposure to human handling, a new physical environment, a different diet, and new social interactions can result in reduced feed intake, decreased growth, and an increased incidence of disease (Jones et al., 2012; McLamb et al., 2013). The response of the intestinal mucosa to weaning is a particularly important concern (Smith et al., 2010). As an immature tissue in the young pig, it suffers some degree of inflammation and dysfunctionality in response to the new antigens, dietary components, and social stressors (Boudry et al., 2004; Pié et al., 2004; Li et al., 2019).

Feed has tremendous potential to improve the weaning transition because it is the most practical means of delivering substances with beneficial nutritional and functional properties to the pig. Although dietary antibiotics have been effectively used to control and prevent diseases in nursery pigs, antibiotic resistance and consumer pressure have demanded a reduction in their use. Therefore, the evaluation of products and dietary components with functional and nutritional properties is increasingly important.

Lactose (LA) is a common carbohydrate in nursery diets as it provides a familiar source of available carbohydrate and induces the production of volatile fatty acids (VFA) in the large intestine (Pierce et al., 2006). On the other hand, a prototype *Lactobacillus Acidophilus* fermentation product (FP; Diamond V, Cedar Rapids, IA) is believed to provide metabolites that enhance intestinal health and the establishment of commensal microorganisms.

The objective of this study was to identify and characterize the beneficial effects of LA and the FP in nursery pig diets with an emphasis on intestinal function. We hypothesized that LA and FP would ameliorate some of the adverse effects of the weaning transition by promoting digestion and decreasing markers of intestinal dysfunction.

## MATERIALS AND METHODS

All experimental procedures adhered to guidelines for the ethical and humane use of animals for research according to the Guide for the Care and Use of Laboratory Animals (FASS, 2010) and were approved by the Institutional Animal Care and Use Committee at Iowa State University (number 7-15-8049-S).

### Animals Housing and Experimental Design

A total of 28 barrows [5.22 ± 0.15 kg body weight (BW); the progeny of C22 or C29 sows × 337 terminal sires; PIC Inc., Hendersonville, TN] were blocked by initial BW (seven blocks) and randomly assigned to individual metabolism crates within block. Crates were randomly assigned to one of four dietary treatments (*n* = 7 pigs per treatment) corresponding to a 2 × 2 factorial design: with (LA+; 15% inclusion) or without (LA−) LA and with (FP+) or without (FP−) the prototype FP (1 g of FP per kilogram of diet; Diamond V, Cedar Rapids, IA).

Diets were manufactured in mash form at the Swine Nutrition Farm feed mill (Iowa State University; Ames, IA; Table 1). Dietary levels of amino acids, vitamins, and minerals were set to meet the nutrient requirements of nursery-aged pigs (NRC, 2012). Titanium dioxide was added at 0.4% to all diets as an indigestible marker.

**Table 1.** Ingredient composition of the experimental diets*

	LA−		LA+	
Item	FP−	FP+	FP−	FP+
Corn	65.73	65.63	50.58	50.48
Soybean meal	20.00	20.00	20.00	20.00
Fish meal	3.72	3.72	3.72	3.72
Casein	5.00	5.00	5.00	5.00
Lactose	—	—	15.00	15.00
Soybean oil	1.50	1.50	1.50	1.50
_L_-Lys HCl	0.40	0.40	0.43	0.43
_DL_-Met	0.15	0.15	0.20	0.20
_L_-Thr	0.15	0.15	0.18	0.18
_L_-Trp	0.01	0.01	0.02	0.02
Monocalcium phosphate 21%	1.14	1.14	1.20	1.20
Limestone	0.90	0.90	0.88	0.88
NaCl	0.50	0.50	0.50	0.50
Vitamin premix†	0.25	0.25	0.25	0.25
Trace mineral premix‡	0.15	0.15	0.15	0.15
Prototype FP	—	0.10	—	0.10
Titanium dioxide	0.40	0.40	0.40	0.40

*LA−: diets without lactose added; LA+: diets with 15% of lactose added; FP−: diets without the prototype FP (Diamond V Mills, Cedar Rapids, IA) added; FP+: diets with 0.1% of prototype FP added (1 g of FP per kilogram of diet; Diamond V Mills, Cedar Rapids, IA) added.

†Provided per kilogram of diet: 7,600 IU of vitamin A; 875 IU of vitamin D3; 62 IU of vitamin E; 3.7 mg of menadione (to provide vitamin K); 61 μg of vitamin B12; 14 mg of riboflavin; 34 mg of d-pantothenic acid; and 70 mg of niacin.

‡Provided per kilogram of diet: 165 mg of Fe (ferrous sulfate); 165 mg of Zn (zinc sulfate); 39 mg of Mn (manganese sulfate); 2 mg of Cu (cooper sulfate); 0.3 ppm of I (calcium iodate); and 0.3 ppm of Se (sodium selenite).

Pigs were housed in a controlled environment facility. Each metabolism crate (0.53 × 0.71 m) was equipped with a fully slatted floor, a stainless-steel feeder, and a cup drinker. Pigs had ad libitum access to feed and water during the entire experimental period (15 d).

### Sample Collection

To measure in vivo gut permeability, all pigs were fasted for 6 h and then orally dosed on day 5 with a solution of 0.300 g lactulose/kg BW and 0.030 g mannitol/kg BW (Sigma-Aldrich, St Louis, MO). Urine was collected for the following 12 h in a plastic jug located under the crate after the administration of the lactulose/mannitol solution. Before urine collections, five drops of chlorhexidine gluconate 20% (weight/volume; Sigma-Aldrich, St Louis, MO) were added to each jug to inhibit bacterial activity. After collection, total urine output was weighed, homogenized, and filtered using fiber glass wool; a 3-mL aliquot of urine sample was collected and stored at −20 °C for later analysis.

In support of determining digestibility and nitrogen (N) balance, and to ensure the acquisition of samples fully representative of the total batch, 10 feed samples were collected from the feed mill at the time of diet preparation; these samples were thoroughly homogenized, pooled into one subsample, and ground through a 1-mm screen in a Retsch grinder (Model ZM1, Retsch Inc., Newton, PA). Total fecal output was collected twice daily from day 5 to 8 and day 10 to 13 and stored at −20 °C. Once collected, fecal samples were thawed, homogenized, subsampled, dried in an oven at 65 °C to constant weight (Jacobs et al., 2011), and ground through a 1-mm screen in a Wiley grinder (Model ED-5, Thomas Scientific Inc., Swedesboro, NJ). Ground fecal and feed samples were stored in plastic bags in desiccator cabinets until assays were completed.

For N balance, total urine output was collected twice daily during days 10–13 (96 h) into plastic jugs containing 5 mL of 6 *N* hydrochloric acid, added before each collection to minimize N losses due to volatilization of ammonia-N. Total urine output was weighed and stored at −20 °C. At the end of the collection period, urine was homogenized and filtered prior to N analysis. Urine samples were aliquoted into 250-mL plastic bottles and stored at −20 °C until chemical analyses were performed. In the determination of N balance, only the days 10–13 fecal samples were utilized to correspond with the urine samples as described above.

All pigs were euthanized on day 15 by captive bolt stunning followed by exsanguination. After euthanasia, each pig was dissected and the entire intestinal tract was removed. Samples (~20 cm sections long) of the proximal jejunum (taken 100 cm from the pyloric sphincter), the distal ileum (taken 30 cm from the ileocecal valve), and the mid colon were removed and gently flushed with ice-cold Krebs buffer. One segment of the jejunum and ileum samples were snap-frozen in liquid N and stored at –80 °C until further analysis. Additionally, subsamples of jejunum, ileum, and colon were fixed for 24 h in 10% neutral buffered formalin and then transferred to 75% alcohol for later morphology and histochemistry analysis. Approximately 20 mL of intestinal contents from the mid-section of the colon were snap-frozen in liquid N and stored at –80 °C.

### Digestibility, N Balance Analysis, and Urinary Lactulose and Mannitol

Feed, fecal, and urine samples were analyzed at the Monogastric Nutrition Laboratory (Iowa State University, Ames, IA). Feed (Table 2) and fecal samples were assayed for dry matter (DM; method 930.15; AOAC, 2007). Gross energy (GE) was determined using a Parr model 6200 isoperibolic bomb calorimeter (Parr Instrument Co., Moline, IL); benzoic acid (6,318 kcal GE/kg; Parr Instruments, Moline, IL) was used as a standard for calibration and was determined to contain 6,319 ± 1 kcal GE/kg. Titanium dioxide was determined colorimetrically using a Synergy 4 spectrophotometer (BioTek, Winooski, VT) according to the method of Leone (1973). Additionally, feed samples were assayed for acid hydrolyzed ether extract (AEE; method 2003.06; AOAC International, 2007) using a SoxCap SC 247 hydrolyzer and a Soxtec 255 semiautomatic extractor (FOSS North America, Eden Prairie, MN). The N content of the feed, fecal, and urine samples was determined by thermocombustion (method 990.03; AOAC International, 2007; Leco TruMac N, LECO Corporation, St. Joseph, MI). The standard for calibration was Ethylenediaminetetraacetic acid (9.56% N; Leco Corporation, St. Joseph, MI) and determined to contain 9.56 ± 0.01% N). Urinary lactulose and mannitol concentrations were determined by High-performance liquid chromatography with known standard solutions according to the method described by Kasangra et al. (2003).

**Table 2.** Chemical composition of the experimental diets, as-fed basis*,†

	LA−		LA+	
Item	FP−	FP+	FP−	FP+
Analyzed chemical composition				
DM, %	88.88	88.76	90.75	90.92
GE Mcal/kg	3.92	3.92	3.94	3.90
AEE, %	4.71	4.70	4.41	4.43
CP, %	21.00	21.06	20.03	20.28
Titanium dioxide, %	0.40	0.41	0.42	0.41
Calculated chemical composition				
SID of AA, %				
Lys	1.44	1.44	1.44	1.44
Thr	0.84	0.84	0.84	0.84
Met	0.55	0.55	0.57	0.57
Met + Cys	0.79	0.79	0.79	0.79
Trp	0.24	0.24	0.24	0.24
Ile	0.82	0.82	0.79	0.79
Val	0.96	0.96	0.92	0.92
Ca, %	0.82	0.82	0.82	0.82
Total P, %	0.70	0.70	0.67	0.67
STTD of P, %	0.42	0.42	0.42	0.42

AA, Amino acids; AEE, acid hydrolyzed ether extract; CP, crude protein; SID, standardized ileal digestibility; STTD, standardized total tract digestible.

*LA−: diets without lactose added; LA+: diets with 15% of lactose added; FP−: diets without the prototype FP (Diamond V Mills, Cedar Rapids, IA) added; FP+: diets with 0.1% of the prototype FP (1 g of FP per kilogram of diet) added.

†AA levels, phosphorus, STTD phosphorus, and calcium were calculated from NRC (2012); all other values were analyzed.

### Intestinal Morphology and Histochemistry Analysis

Fixed jejunum, ileum, and colon samples were prepared and stained at the Veterinary Diagnostic Laboratory (Iowa State University, Ames, IA). Each sample was sliced into 5-μm sections. The jejunum and ileum sections were stained using the hematoxylin and eosin procedure, whereas colon samples were stained with the alcian blue-periodic acid-Schiff (AB-PAS) procedure (Mowry, 1963) to distinguish between neutral and acidic mucin types in colon crypts. The neutral mucin was stained magenta, and acidic mucin was stained blue. The mixture of neutral-acidic mucins showed purple, magenta-purple, or blue-purple colors in goblet cells.

Images of intestinal mucosa were captured using a light microscope (DMI3000 B Inverted Microscope, Leica Microsystems, Bannockburn, IL) with an attached camera (12-bit QICAM Fast 1394, QImaging, Surrey, BC, Canada). Individual images of villi and crypts were taken using Q-capture Pro 6.0 software (QImaging, Surrey, BC) and measured using Image-Pro Plus 7.0 (Media Cybernetics, Bethesda, MD). Images were measured using Image J software, version 1.48s (Rasband W, National Institutes of Health, MD). The villus height was measured from the tip of the villus to the crypt–villus junction, and the crypt depth was measured from the crypt–villus junction to the base of the crypt. At least 10 well-defined villi and associated crypts from each sample of each intestinal segment were measured; averages were then calculated and reported as one number per pig. The mucin area was calculated as a percentage of the total mucosal tissue area in each image.

### Enzyme Activity and Secretory Immunoglobulin A (S-IgA)

Mucosal scrapings (~0.5 g) of the jejunum and ileum were added to 4.5 mL of Phosphate-buffered saline buffer containing a protease inhibitor cocktail (SKU, P8340; Sigma-Aldrich, St. Louis, MO) and triton (0.1%). The resulting solution was homogenized and centrifuged at 10,000 × *g* for 15 min at 4 °C. The supernatant was extracted and stored in aliquots. The total protein concentration of hydrolyzed mucosa scrapings was quantified using a Pierce Bicinchoninic Acid Protein Assay Kit (BCA; Thermo Scientific, Woltham, MA). Disaccharidase activity was determined according to Dahlqvist (1964) using LA, maltose, and sucrose as substrates. Alkaline phosphatase activity was determined using a porcine alkaline phosphatase assay kit (ABCAM, Cambridge, MA). Enzyme activity was expressed as micromole of hydrolyzed substrate per minute per gram of tissue protein. The concentration of secretory IgA was determined using a porcine IgA ELISA kit (E101-102; Bethyl Laboratories, Inc., Montgomery, TX) following the manufacturer’s directions.

### RNA Extraction and Real-Time PCR

RNA was extracted from frozen jejunal and ileal tissues according to the Trizol protocol (Invitrogen, Grand Island, NY). The purity and quantity of RNA were determined by spectrophotometry using a Nanodrop 1000 instrument (NanoDrop Technologies, Rockland, DE). The purity was assessed by determining the ratio of the absorbance at 260 and 280 nm (all samples had absorbance ratios above 1.8). One microgram of RNA was transcribed in a reaction combining genomic DNA elimination using a commercially available kit (Quantitect reverse transcription kit; Qiagen Inc., Valencia, CA). The resulting complementary DNA was quantified using a NanoDrop 1000 instrument and applied to quantitative real-time PCR (qPCR). The qPCR was completed using a BioMark HD system (Fluidigm Corporation, San Francisco, CA). No reverse transcriptase control samples were included in the extraction process to assure no genomic DNA contamination. Complementary DNA was used for specific target amplification using the TaqMan PreAmp Master Mix (Life Technologies, Carlsbad, CA) and loaded onto Fluidigm’s Dynamic Array Integrated Fluidic Circuits according to Fluidigm’s EvaGreen DNA binding dye protocols. Gene symbols and primer sequences are listed in Table 3. One 48.48 Dynamic Array, Integrated Fluidic Circuit plate, was used to analyze mRNA abundance of selected genes in porcine jejunal and ileal tissues. To select an endogenous reference gene, ribosomal protein L19 (RPL19) was included in the qPCR array.

**Table 3.** Primers used for quantitative reverse transcription-PCR

		Primer sequence, 5ʹ→3 ʹ		
Gene name	Accession number	Sense (forward)	Anti-sense (reverse)
RPL19	AF435591	AACTCCCGTCAGCAGATCC	AGTACCCTTCCGCTTACCG
OCLN	NM_001163647	TCGTCCAACGGGAAAGTGAA	ATCAGTGGAAGTTCCTGAACCA
CLDN3	NM_001160075	TTGCATCCGAGACCAGTCC	AGCTGGGGAGGGTGACA
IL-6	AF518322	GGCTGTGCAGATTAGTACC	CTGTGACTGCAGCTTATCC
IL-10	L20001	TGGGTTGCCAAGCCTTGT	GCCTTCGGCATTACGTCTTC
IL-17a	AB102693	CCAGACGGCCCTCAGATTAC	CACTTGGCCTCCCAGATCAC
IL-22	AY937228	AAGCAGGTCCTGAACTTCAC	CACCCTTAATACGGCATTGG
TNFα	X54859	AACCCTCTGGCCCAAGGA	GGCGACGGGCTTATCTGA

### VFA Concentration

In preparation for VFA analysis, 1.0 g of colon contents were mixed with 2.5 g of purified water and 1 mL was extracted from the homogenized solution. Then 0.2 mL of 25% metaphosphoric acid (used to deprotonize the samples) and 0.1 mL of 4-methylvaleric acid (as an internal standard) were added to the extracted homogenate. These samples were centrifuged at 12,000 × *g* for 25 min, and the supernatant was removed for analysis. Volatile fatty acids were determined using a model 3900 gas chromatograph fitted with a CP 8400 automatic injector (Varian Analytical Instruments, Walnut Creek, CA) using a 30 m, 0.25 mm, and 0.25 μm column (DB-FFAP, Agilent, Santa Clara, CA). Helium was used as carrier gas. Purified VFA samples (Sigma-Aldrich, St. Louis, MO) were used for the identification of VFA peaks.

### Calculations

Individual pig weights and feed disappearance were measured on days 0 and 14 to calculate average daily gain (ADG), average daily feed intake (ADFI), and gain to feed ratio (G:F). Apparent total tract digestibility (ATTD) of DM, GE, and N was calculated using the following equation (Oresanya et al., 2008):

$$\begin{array}{ll} ATTD,\;\%\;= & \;[100\;\;[100\;\times \;(\%\;TiO2\;in\;feed/\%\;TiO2\;in\;feces)\;\\ & \times \;(concentration\;of\;component\;in\;feces/\\ & \;concentration\;of\;component\;in\;feed)]] \end{array}$$

N intake (g/d) was calculated by multiplying the percentage of N in the feed (DM basis) by DM feed intake (g/d); N excreted in the urine was calculated by multiplying the average daily urine weight (kg/d) by the average urinary N concentration (%). Nitrogen excreted in feces (g/d) was calculated by multiplying daily N intake minus the product of multiplying N intake times the ATTD of N (%). Total N excretion (g/d) was calculated as the sum of daily N excreted in urine and feces. Finally, N retention was calculated as daily N intake minus daily N excretion. Protein retention was calculated as N retention (g/d) × 6.25 divided by the ADG (g/d). All N balance variables were calculated using data from samples collected on days 10–14.

Lactulose and mannitol recoveries (%) were calculated as the amount of these sugars excreted (g/d) in the urine [calculated as total urine weight (g) times lactulose or mannitol concentration (%)] divided by the amount of lactulose or mannitol given (g/d) orally times 100. Lactulose:mannitol ratio was calculated by dividing lactulose recovery by mannitol recovery (Musa et al., 2019).

Plates for PCR analysis were balanced so that an equal number of treatments were represented on each plate. Additionally, a pooled control sample representative of all treatment groups was run on each gene as an internal control. Normalized expression (ΔCt) for each sample was determined using RPL19 as an endogenous housekeeping gene. The average normalized expression of the pooled control sample was used as the calibrator to calculate relative gene expression (Livak and Schmittgen, 2001). For each sample, relative expression was calculated as 2^–ΔΔCt^, in which ΔΔCt represents ΔCt sample − ΔCt calibrator (Livak and Schmittgen, 2001).

### Statistical Analysis

Data were analyzed using the following model:

$$\begin{array}{l} y_{ijkl}=\;\;\;\mu +\tau_{i}+\lambda_{j}+{\left(\tau \lambda \right)}_{ij}+\delta_{k}+\;\;\;\epsilon_{ijkl} \end{array}$$

where *y*_*ijkl*_ represents the observed value, μ is the overall mean, τ represents the fixed effect of LA [*i* = 1, 2 (LA− and LA+, respectively)], λ represents the fixed effect of FP [*j* = 1, 2 (FP− and FP+, respectively)], τλ represents the interaction between LA and FP, δ represents the random effect of block (*k* = 1–7), and ϵ is the random error associated with *y*_*ijkl*_ (*l* = 1–7) assuming δ ~Ν (0, ) and ϵ ~Ν (0, ).

The pig was the experimental unit for all analyses. The UNIVARIATE procedure of SAS version 9.3 (SAS Inst., Inc., Cary, NC) was used to verify the normality and homogeneity of the residual variance from the reported models. Jejunal and ileal mucosa S-IgA, mannitol, and lactulose recovery and lactulose:mannitol ratio variables were analyzed and reported after log-transformation to satisfy normality. The models were analyzed using the MIXED procedure of SAS. Differences were considered significant with *P*-values ≤0.050, and *P*-values between 0.050 and 0.100 were considered trends.

## RESULTS

### Growth Performance, Digestibility, and N Balance

Evaluating the impact of dietary treatments on growth performance in metabolism crates is difficult due to the typically small number of animals and the conditions that differ substantially from normal housing. Nonetheless, it is useful to do so to provide an understanding of the state of the animal under which other measurements were recorded.

No interactions between LA and FP were observed for initial BW, final BW, growth performance, digestibility, or N balance variables. Therefore, the results of these variables are presented as the main effects. By design, the initial BW was not affected by the addition of LA or FP (Table 4). Similarly, final BW (day 14) and ADG were not affected by the addition of LA or the FP. Although there was no effect of FP on ADFI, pigs fed LA had increased ADFI (*P* = 0.017).

**Table 4.** Effect of LA and a prototype *Lactobacillus acidophilus* FP on growth performance of nursery pigs*,†

	LA		FP			*P-*value	
Item	−	+	−	+	SEM	LA	FP
Day 0 BW, kg	5.23	5.23	5.22	5.23	0.06	0.744	0.334
Day 14 BW, kg	7.23	7.78	7.70	7.30	0.26	0.105	0.216
ADG, kg	0.148	0.189	0.184	0.153	0.018	0.102	0.205
ADFI, kg	0.180	0.229	0.218	0.190	0.016	0.017	0.150
G:F	0.787	0.809	0.822	0.774	0.043	0.731	0.445

*A total of 28 barrows (5.22 ± 0.15 kg BW) assigned to individual metabolism crates for 15 d (seven pigs per treatment).

†LA−: diets without lactose added; LA+: diets with 15% of lactose added; FP−: diets without the prototype FP (Diamond V Mills, Cedar Rapids, IA) added; FP+: diets with 0.1% of the prototype FP (1 g of FP per kilogram of diet) added.

The addition of LA improved the ATTD of DM and GE (*P* = 0.014 and *P* = 0.028, respectively; Table 5). However, the addition of the FP did not affect the ATTD of DM or GE. There were no effects of LA on the ATTD of N. However, the addition of the FP tended to increase the ATTD of N (*P* = 0.090).

**Table 5.** Effect of LA and a prototype *Lactobacillus acidophilus* FP on ATTD of DM, GE, and N in nursery pigs*,†

	LA		FP			*P-*value	
Item	−	+	−	+	SEM	LA	FP
ATTD of DM, %	88.1	89.2	88.4	88.9	0.4	0.014	0.167
ATTD of GE, %	86.4	87.7	86.7	87.4	0.5	0.028	0.206
ATTD of N, %	83.8	83.6	82.9	84.5	0.9	0.900	0.090

*A total of 28 barrows (5.22 ± 0.15 kg BW) assigned to individual metabolism crates for 15 d (seven pigs per treatment), diets contained titanium dioxide (0.4%) as an indigestible marker, and total fecal output was collected twice daily from day 5 to 8 and day 10 to 13.

†LA−: diets without lactose added; LA+: diets with 15% of lactose added; FP−: diets without the prototype FP (Diamond V Mills, Cedar Rapids, IA) added; FP+: diets with 0.1% of prototype FP (1 g of FP per kilogram of diet) added.

The addition of LA tended to increase N intake (*P* = 0.080), whereas no effect of FP was observed (Table 6). In contrast, the FP tended to decrease N excretion (*P* = 0.060), whereas LA did not affect N excretion. When N excretion was partitioned into fecal or urinary components, the addition of LA increased fecal N excretion (*P* = 0.017), while FP decreased it (*P* = 0.044). The addition of LA decreased urinary N and increased the overall N retention (*P* = 0.006 and *P* = 0.043, respectively). The addition of FP did not affect urinary N excretion or overall N retention, expressed as g/d.

**Table 6.** Effect of LA and a prototype *Lactobacillus acidophilus* FP on N balance of nursery pigs*,†

	LA		FP			*P-*value	
Item	−	+	−	+	SEM	LA	FP
N balance, g/d							
Intake	7.57	9.28	8.80	8.06	0.65	0.072	0.414
Excretion	2.85	2.88	3.09	2.64	0.19	0.901	0.060
Fecal	1.16	1.59	1.55	1.20	0.15	0.017	0.044
Urine	1.68	1.29	1.54	1.44	0.09	0.006	0.468
Retention	4.72	6.41	5.71	5.42	0.56	0.043	0.716
N balance %							
Excretion	38.7	32.1	35.8	35.0	2.1	0.043	0.804
Fecal	15.0	17.3	17.4	14.9	1.1	0.163	0.120
Urinary	23.7	14.8	18.3	20.2	1.7	0.002	0.445
Retention	61.3	67.9	64.2	65.0	2.1	0.043	0.804
Protein retained as % of ADG	15.0	17.0	15.2	16.8	0.6	0.020	0.053

*A total of 28 barrows (5.22 ± 0.15 kg BW) was assigned to individual metabolism crates for 15 d (seven pigs per treatment); total urine and fecal output was collected twice daily during days 10–13 (96 h). ADG was calculated using weights collected on days 10 and 14.

†LA−: diets without lactose added; LA+: diets with 15% of lactose added; FP−: diets without the prototype FP (Diamond V Mills, Cedar Rapids, IA) added; FP+: diets with 0.1% of the prototype FP (1 g of FP per kilogram of diet) added.

The addition of LA decreased the percentage of N excreted. There was no effect of LA on the percentage of N excreted in the feces, but it decreased the percentage of N excreted in the urine (*P* = 0.002). As a result, the addition of LA increased the percentage of N retained (*P* = 0.043). The addition of FP had no effect on the total percentage of N excreted or the percentage of N excreted in the urine and the feces. Consequently, the addition of FP had no effect on the percentage of N retained. The addition of LA increased the protein retained as a percentage of ADG (*P* = 0.020), whereas FP tended to increase the protein retained as a percentage of ADG (*P* = 0.053). The protein retained expressed as a percentage of ADG is a useful standard to validate N retention data—it should be in the range of 15–18% for pigs of this age—and is also a very good indicator of the lean gain in the pigs.

### Intestinal Morphology, Mucin Staining Area, and Concentration of S-IgA

The morphology of the small intestine was affected by the interaction between LA and FP, so individual treatment means are presented. In the jejunum, pigs fed LA+FP− had increased villus height compared with those fed LA+FP+ and LA−FP−, whereas LA-FP+ was intermediate (Table 7; interaction *P* = 0.034). In the terminal ileum, pigs fed LA−FP+ and LA+FP− had increased villus height compared with those fed LA−FP−, whereas LA+FP+ was intermediate (interaction *P* = 0.007). Although neither LA nor FP altered villus:crypt ratio in the jejunum, an interaction between LA and FP affected villus:crypt ratio in the ileum; pigs fed LA−FP+ and LA+FP− had increased villus:crypt ratio compared with those fed LA−FP−, whereas LA+FP+ was intermediate (interaction *P* = 0.007). Neither LA nor FP affected crypt depth at the jejunum or at the ileum.

**Table 7.** Effect of LA and a prototype *Lactobacillus acidophilus* FP on intestinal morphology, S-IgA, and mucins of nursery pigs*,†

	LA−		LA+			*P-*value		
Item	FP−	FP+	FP−	FP+	SEM	LA	FP	LA × FP
Jejunum								
Villus height, μm	275^b^	320^ab^	390^a^	307^b^	28	0.089	0.511	0.034
Crypt depth, μm	313	338	343	326	21	0.677	0.848	0.321
Villus: crypt	0.88	1.00	1.15	0.95	0.11	0.321	0.682	0.160
S-IgA, (log) μg/mg of protein	3.04^a^	2.41^b^	2.59^b^	2.64^b^	0.14	0.637	0.065	0.005
Ileum								
Villus height, μm	266^b^	341^a^	378^a^	317^ab^	25	0.065	0.780	0.007
Crypt depth, μm	262	247	263	274	16	0.332	0.891	0.391
Villus: crypt	1.02^b^	1.43^a^	1.48^a^	1.19^ab^	0.12	0.351	0.591	0.007
S-IgA, (log) μg/g of protein	2.62	2.76	2.71	2.70	0.12	0.939	0.610	0.520
Colon								
Crypt depth, μm	435	395	440	420	24	0.502	0.189	0.645
Goblet cells/100 μm crypt	5.8	6.2	6.0	5.3	0.7	0.720	0.782	0.263
Acid mucins, %	11.4	11.0	12.8	7.3	1.4	0.437	0.053	0.087
Mixed mucins, %	12.7	10.7	13.7	11.8	2.0	0.597	0.318	0.979
Neutral mucins, %	2.6^a^	1.6^b^	2.1^ab^	3.1^a^	0.4	0.175	0.938	0.010

^a,b^Means with different superscripts significantly differ (*P* < 0.050).

*A total of 28 barrows (5.22 ± 0.15 kg BW) assigned to individual metabolism crates for 15 d (7 pigs/treatment), intestinal tissues, and digesta were collected after euthanasia.

†LA−: diets without lactose added; LA+: diets with 15% of lactose added; FP−: diets without the prototype FP (Diamond V Mills, Cedar Rapids, IA) added; FP+: diets with 0.1% of the prototype FP (1 g of FP per kilogram of diet) added.

In the mid colon, there were no effects of LA or FP on crypt depth, the number of goblet cells per crypt, or the percentage of acid or mixed mucins. However, there was an interaction between LA and FP on the percentage of neutral mucins: pigs fed LA−FP− and LA+FP+ had an increased percentage of neutral mucins compared with those fed LA−FP+, whereas LA+FP− was intermediate (interaction *P* = 0.010).

The concentration of S-IgA was affected by the interaction of LA and FP. Pigs fed LA−FP− had increased levels of mucosal S-IgA compared with the other three treatments (interaction *P* = 0.005). However, neither LA nor FP impacted the concentration of S-IgA at the terminal ileum.

### Small Intestine Permeability, Enzymatic Activity, and Transcript Abundance of Tight Junction Proteins and Cytokines

There were no main effects or interactions of LA or FP on mannitol recovery, lactulose recovery, or in the lactulose:mannitol ratio (Table 8). Similarly, activities of lactase, sucrase, maltase, or alkaline phosphatase were not affected by the addition of LA or FP (Table 9). Likewise, gene transcript abundance of tight junction protein genes [occludin (OCLN) and claudin-3 (CLDN3); Table 10] in the jejunum and terminal ileum and ileal cytokines [tumor necrosis factor alpha (TNFα), interleukin-6 (IL-6), IL-10, IL-17, and IL-22] were not affected by the addition of LA or FP.

**Table 8.** Effect of LA and a prototype *Lactobacillus acidophilus* FP on small intestinal permeability at day 5 postweaning*,†

	LA−		LA+			*P-*value		
Item	FP−	FP+	FP−	FP+	SEM	LA	FP	LA × FP
Mannitol recovery, (log) %	1.41	1.29	1.37	1.51	0.12	0.435	0.942	0.281
Lactulose recovery, (log) %	−0.14	−0.16	−0.38	−0.17	0.16	0.432	0.553	0.479
Lactulose: mannitol (log)	−1.54	−1.55	−1.66	−1.66	0.16	0.530	0.977	0.956

*A total of 28 barrows (5.22 ± 0.15 kg BW) assigned to individual metabolism crates for 15 d (seven pigs per treatment). Intestinal permeability was assessed by providing each pig a solution of 0.300 g lactulose/kg BW and 0.030 g mannitol/kg BW (Sigma-Aldrich, St Louis, MO). Urine was collected for the following 12 h after the administration of the lactulose/mannitol solution.

†LA−: diets without lactose added; LA+: diets with 15% of lactose added; FP−: diets without the prototype FP (Diamond V Mills, Cedar Rapids, IA) added; FP+: diets with 0.1% of the prototype FP (1 g of FP per kilogram of diet) added.

**Table 9.** Effect of LA and a prototype *Lactobacillus acidophilus* FP on the enzymatic activity at the jejunum*

	LA−		LA+			Contrast *P-*value†		
Item	FP-	FP+	FP-	FP+	SEM	LA	FP	LA × FP
Activity, U/mg protein								
Lactase	16.6	10.6	15.7	14.7	3.6	0.976	0.335	0.222
Sucrase	20.9	25.5	31.2	24.3	5.1	0.387	0.847	0.511
Maltase	182.7	190.7	206.2	228.4	22.4	0.116	0.427	0.708
Alkaline phosphatase	60.7	60.6	65.4	53.1	6.4	0.833	0.340	0.348

*A total of 28 barrows (5.22 ± 0.15 kg BW) assigned to individual metabolism crates for 15 d (seven pigs per treatment); jejunal tissue was collected at day 15 after euthanasia.

†LA−: diets without lactose added; LA+: diets with 15% of lactose added; FP−: diets without the prototype FP (Diamond V Mills, Cedar Rapids, IA) added; FP+: diets with 0.1% of the prototype FP (1 g of FP per kilogram of diet) added.

**Table 10.** Effect of LA and a prototype *Lactobacillus acidophilus* FP on RNA abundance of tight junction proteins and cytokines in the jejunum and ileum*,†,‡

	LA−		LA+			*P-*value		
Item	FP−	FP+	FP−	FP+	SEM	LA	FP	LA × FP
Jejunum								
OCLN	1.23	1.44	2.08	1.20	0.37	0.394	0.352	0.139
CLDN3	2.33	1.56	1.63	1.76	0.40	0.550	0.448	0.285
Ileum								
OCLN	1.67	1.49	1.10	1.09	0.36	0.223	0.892	0.569
CLDN3	1.59	1.48	0.87	1.28	0.36	0.565	0.873	0.353
TNFα	1.03	1.37	1.14	1.36	0.27	0.797	0.146	0.727
IL-6	0.61	0.79	1.17	0.99	0.24	0.102	0.839	0.324
IL-10	0.87	1.04	1.23	1.15	0.26	0.264	0.801	0.545
IL-17	0.80	1.03	1.15	0.85	0.18	0.618	0.872	0.137
IL-22	1.08	1.35	1.14	0.77	0.54	0.900	0.543	0.238

*All values indicate relative expression of genes. Normalized expression (ΔCt) for each sample was determined using RPL19 as an endogenous control gene. The average normalized expression of the pooled control sample was used as the calibrator to calculate relative gene expression. For each sample, relative expression was calculated as 2^−ΔΔCt^, in which ΔΔCt represents ΔCt sample − ΔCt calibrator (Livak and Schmittgen, 2001).

†A total of 28 barrows (5.22 ± 0.15 kg BW) assigned to individual metabolism crates for 15 d (seven pigs per treatment); intestinal tissue was collected at day 15 after euthanasia.

‡LA−: diets without lactose added; LA+: diets with 15% of lactose added; FP−: diets without the prototype FP (Diamond V Mills, Cedar Rapids, IA) added; FP+: diets with 0.1% of the prototype FP (1 g of FP per kilogram of diet) added.

### VFA Concentrations in the Colon

Although LA had no effect, the addition of FP decreased the concentration of acetic acid in the colon (Table 11; *P* = 0.022). Neither LA nor FP affected the concentration of propionic acid. Pigs fed LA tended to increase butyric acid concentration (*P* = 0.062), but there was no effect when adding FP. There were no effects of LA or FP on the concentration of branched-chain fatty acids (isobutyric, isovaleric, and valeric acid). Pigs fed LA tended to increase total VFA (*P* = 0.099), whereas the addition of FP decreased total VFA concentration in colonic contents (*P* = 0.048).

**Table 11.** Effect of LA and a prototype *Lactobacillus acidophilus* FP on volatile fatty acid concentrations in colonic contents*,†

	LA−		LA+			*P-*value		
Item	FP−	FP+	FP−	FP+	SEM	LA	FP	LA × FP
Concentration μM/g								
Acetic acid	63.77	49.76	71.53	58.17	6.07	0.292	0.022	0.767
Propionic acid	24.14	18.48	26.86	23.13	3.00	0.232	0.132	0.749
Butyric acid	15.16	14.56	20.60	20.19	2.81	0.062	0.858	0.974
Isobutyric acid	2.21	2.34	3.21	2.07	0.44	0.522	0.201	0.129
Isovaleric acid	3.46	3.69	4.97	3.23	0.71	0.468	0.303	0.184
Valeric acid	3.30	3.21	4.65	4.36	0.81	0.173	0.676	0.819
Total	115.1	92.1	131.8	111.2	10.3	0.099	0.048	0.910
Distribution, %								
Acetic acid	58.1	55.1	54.3	52.8	3.2	0.330	0.471	0.810
Propionic acid	20.8	19.6	20.3	20.7	1.5	0.850	0.804	0.591
Butyric acid	13.2	15.1	15.6	17.7	1.6	0.140	0.227	0.945
Isobutyric acid	1.9^y^	2.6^x^	2.4^xy^	1.9^y^	0.3	0.729	0.761	0.069
Isovaleric acid	3.0^y^	4.1^x^	3.8^xy^	3.0^y^	0.5	0.740	0.842	0.099
Valeric acid	2.9	3.5	3.7	3.9	0.7	0.660	0.405	0.559

^x,y^Means with different superscripts tend to differ (*P* < 0.100).

*A total of 28 barrows (5.22 ± 0.15 kg BW) assigned to individual metabolism crates for 15 d (seven pigs per treatment); intestinal tissue was collected at day 15 after euthanasia.

†LA−: diets without lactose added; LA+: diets with 15% of lactose added; FP−: diets without the prototype FP (Diamond V Mills, Cedar Rapids, IA) added; FP+: diets with 0.1% of the prototype FP (1 g of FP per kilogram of diet) added.

When VFAs were looked at as proportions of the total concentration, the percentages of acetic, propionic, butyric, and valeric acid were not affected by the addition of LA or FP. However, LA and FP tended to interact for the percentages of branched-chain fatty acids; pigs fed LA−FP+ tended to have increased percentages of isobutyric and isovaleric acid compared with LA−FP− and LA+FP+, whereas LA+FP− was intermediate (interaction *P* = 0.069 and *P* = 0.099, respectively).

## DISCUSSION

One of the most important strategies to ameliorate the impact of weaning stress is the use of specialty ingredients and feed additives targeted to possess nutritional, as well as functional, properties. The primary target of these functional properties is the gastrointestinal tract. The first experimental approach herein was to evaluate the effect of LA and FP on digestibility and N retention while at the same time monitoring feed intake and growth performance. These would represent key outcomes that would potentially be improved if negative effects of weaning are reduced; more specifically, the consumption of specialty ingredients may ameliorate stress on the intestinal tract.

Postweaning anorexia has profound negative effects on the growth and health of nursery pigs (Spreeuwenberg et al., 2001) and is the main factor affecting subsequent growth performance (Jones et al., 2012). Therefore, dietary compounds that stimulate appetite are highly desirable. Lactose is the primary dietary carbohydrate consumed before weaning (~5% in sow milk; Rosero et al., 2015) and is believed to be highly palatable to the nursery pig. Results of this experiment and other research (Tokach et al., 1989; Kim et al., 2010; Tran et al., 2012; Pierce et al., 2006) support the use of LA in the starter diet to enhance feed intake during the postweaning period.

In addition to stimulating appetite, nursery diets are designed to be highly digestible. This is because feed intake and gut capacity of weaned pigs is limited (Dong and Pluske, 2007). Additionally, the digestibility of nutrients is a key determinant of the growth response of nursery pigs after weaning (Jones and Patience, 2014). The addition of LA increased the digestibility of DM and GE. These same results were reported by Jin et al. (1998) by adding 20% of LA to nursery pigs and by Pierce et al. (2005) feeding increased levels of LA (0–11%) to finishing pigs. Compared with corn, LA has a much simpler chemical and physical structure that is readily available to be cleaved by lactase at the brush border of the small intestine and absorbed by the enterocytes as galactose and glucose. Additionally, LA escaping digestion is rapidly utilized by bacterial populations in the distal small intestine, as well as in the large intestine (Bach Knudsen, 2012). Thus, the results of this experiment confirm that LA contributes to the objective of making nursery diets highly digestible by providing readily usable energy and an easily fermentable substrate.

Protein deposition is the most valued component of the total weight gain of the nursery pig (de Vries and Kanis, 1994; Colina et al., 2010). In this experiment, the addition of LA increased overall N retention, the percentage of N retained, and the protein retained as the percentage of the ADG. The increase in overall N retention can be explained largely by the increase in feed intake in the LA-fed pigs since nutrient supply is the main factor limiting protein deposition in nursery pigs (Van Milgen et al., 2000). Interestingly, pigs fed LA not only had greater protein deposition but were more efficient in using N toward protein deposition. These results agree with those obtained by Pierce et al. (2005), who fed a LA supplement to finishing pigs. Interestingly, they also observed that LA improves N utilization by decreasing the percentage of N excreted in the urine and not through an increase in N digestibility.

Independent of the responses to LA, the addition of FP tended to increase the digestibility of N and the protein retained as a percentage of ADG; this suggested that the mode of action of FP may be related to improving the ability of the pig to digest dietary protein.

Overall, the results of the data on nutrient digestibility and N balance, within the context of growth performance, suggest that LA plays an important role in nourishing the weaned pig by improving the digestibility of DM and GE and N retention, as well as improving feed intake. The addition of FP had a more modest impact on the digestibility of N, with no detectable improvement in growth.

The second experimental approach was to explore the effects of LA and FP on specific markers of intestinal function. Recovery from weaning stress by the intestinal tissue has been described to occur in two phases: the first is an acute phase, lasting 2–4 d, in which the major dysfunctional changes occur, including the disruption of barrier function and immune activation (including inflammation; Pié et al., 2004; Smith et al., 2010). The subsequent adaptive phase, lasting about 2 wk after the acute phase, is associated with tissue recovery (Montagne et al. 2007). The length of the acute phase and a proper recovery of the intestinal tissue after weaning are key determinants of the performance and the health parameters of nursery pigs and potentially their performance in subsequent growth stages. Therefore, the addition of functional products and specialty ingredients targeted to ameliorate the weaning transition should help the pig recover from one or more of these dysfunctional changes. One of the most important targets is the speed of barrier function restitution after weaning (Wijtten et al., 2011). The results of this experiment suggest no effect of LA and FP on the permeability of the small intestine at the end of the acute phase of weaning (day 5). Likewise, no effects on the RNA abundance of CLDN3 and OCLN—essential components of the tight junction complex (Saitou 1997; Niewold, 2015)—or on the mRNA abundance of proinflammatory cytokines in the ileum (IL-6, IL-10, IL-22, and TNFα) were observed in the small intestine at the end of the adaptive phase of weaning (day 14). Proinflammatory cytokines are expected to be upregulated when immune cells are recruited (Renz et al., 2012). No effects of LA on these variables have been found in the literature, but the FP has been shown to decrease proinflammatory mediators (such as TNFα, interferon gamma, and IL-6, IL-8, and IL-1-beta-1) under an LPS challenge (Lee et al., 2016). An alternative explanation of these results is that the weaning stress experienced by these pigs, not associated with clinical signs of disease, was mild (Pié et al., 2004: Montagne et al., 2007), resulting in a limited window of chance for improvement of makers of barrier function and inflammation. However, this explanation is difficult to support since there was no measurement of the stress experienced by these pigs in the first place.

Despite a lack of responses on the barrier function and the inflammation parameters, the results obtained in this experiment showed less S-IgA concentration in the jejunum of pigs fed either LA or FP. The concentration of S-IgA in weaned pigs is the result of an adaptive immunological response to the exposure to luminal immunogenic molecules (Rey et al., 2004; Suzuki and Fargasan, 2008; Brandtzaeg, 2013). Additionally, the increase in S-IgA concentration can be the result of the normal development of the acquired immunity of the weaned pig (McGlone and Pond, 2003). Since the decrease in the concentration of S-IgA in pigs fed LA and FP was observed only in the jejunum, it is likely the result of a localized response of the pigs; there probably was no significant impact of weaning on the intestinal health or the development of the acquired immunity of these pigs. Thus, additional research is needed to determine if LA and FP decrease the growth of antigenic-IgA triggering bacteria probably by inducing the growth of nonpathogenic microorganisms.

Among the multiple changes in gastrointestinal function in the weaning pig, the activity of disaccharidases have been reported to shift during the weaning recovery; lactase activity decreases while maltase activity increases after weaning (Montagne et al., 2007; Tsukahara et al., 2013). In this experiment, lactase activity was expected to be elevated on day 15 in pigs receiving LA due to substrate induction, the consequence of keeping LA in the diet. However, similar lactase activities were observed between the pigs receiving the LA+ and LA− diets. To the knowledge of the authors, there is no specific data reporting substrate induction for lactase activity in nursery pigs. Instead, the mentioned shift in disaccharidase activities has been reported to take place regardless of the presence of LA in the diet (Hedemann et al., 2006; Montagne et al., 2007). Troelsen et al. (1992) and Motohashi et al. (1997) suggested that the decline in lactase activity after weaning is the result of the decrease of the intestinal nuclear factor [NF-LPH1] lactase promoter and the LA expression during the intestinal development process after weaning rather than lack of LA induction in the diet. The current experiment not only supports this rational but also suggests that even with the reduction of lactase activity during the postweaning period, the weaned pig has enough lactase activity to effectively use LA− based on the digestibility and N balance results.

In conclusion, the addition of LA brings important nutritional attributes to nursery diets by improving feed intake, digestibility of DM and GE, and N retention of weaned pigs; however, the functional capacity of LA to improve markers of intestinal function is limited at least under the conditions of this study. On the other hand, the FP showed only a mild increase in the digestibility of N but a limited capacity to improve markers of intestinal function.

*Conflict of interest statement*. The authors declare no real or perceived conflicts of interest.
